# Fostering engagement in EFL listening: how teacher autonomy support mediates the impact of academic emotions

**DOI:** 10.3389/fpsyg.2026.1746522

**Published:** 2026-02-25

**Authors:** Jiayan Zeng, Aonan Peng, Fang Huang, Chenggang Wu

**Affiliations:** 1 School of Teacher Education, Dali University, Dali, China; 2 School of Education, Central China Normal University, Wuhan, China; 3 School of Education, Shanghai International Studies University, Shanghai, China; 4 Institute of Language Sciences, Shanghai International Studies University, Shanghai, China

**Keywords:** academic emotions, anxiety, English listening learning, learning autonomy, learning engagement

## Abstract

This study investigates how academic emotions and autonomy support shape engagement in English as a foreign language (EFL) listening, a skill that receives less research attention than reading, writing, or speaking. Two hundred and nineteen Chinese undergraduates completed scales on academic emotions, perceived autonomy support, and their behavioral and emotional engagement in listening activities. We found that the impact of academic emotions on listening learning engagement presented a complex, multi-path system. The results showed that enjoyment most directly and powerfully enhanced learning engagement, while hopelessness and anger directly diminished affective and behavioral engagement, respectively. Meanwhile, although pride and anxiety did not have a direct effect, they could indirectly be transformed into positive drivers by stimulating students’ perception of teacher autonomy support; conversely, boredom indirectly inhibited engagement by undermining such support. Our findings suggest that comprehension difficulties can sharpen students’ emotional responses, and these feelings, in turn, influence their engagement in listening tasks. This study indicates that an autonomy-supportive learning environment can foster students’ positive emotional experiences and promote their engagement in EFL listening.

## 1 Introduction

While recent research on English as a Foreign Language (EFL) has highlighted the roles of academic emotions and learner autonomy in student engagement (Bordbar, 2021; Murphy et al., 2019; Yousefi and Bordbar, 2017), the focus has predominantly been on reading, writing, and speaking. Listening tasks have often been neglected. This gap is significant, as listening is a crucial skill for EFL learners, who are expected to use it in diverse contexts ranging from casual conversations to academic lectures (Hogan et al., 2014; Rubin, 1994).

Unlike reading and writing, where learners can slow down and revisit materials, listening is characterized by its real-time, transient nature. Although modern technologies like playback controls and transcriptions can offer some review opportunities, the spontaneous flow of spoken input demands immediate cognitive processing, placing a distinct cognitive and emotional load on learners (Graham, 2006; Vandergrift, 2006). Furthermore, processing spoken foreign language input requires learners to decode unfamiliar sound patterns, adapt to varying accents and speech rates, and infer meaning from contextual clues, challenges that are less pronounced or differently manifested in text-based tasks. Consequently, EFL learners often experience heightened intensity in discrete academic emotions, particularly negative activating emotions like anxiety and frustration, which can directly affect their engagement and comprehension.

Despite the importance of listening, the affective dimension of this skill remains underexplored. Emotions such as enjoyment, pride, anxiety, and boredom are known to shape learners’ motivation and outcomes (Pekrun, 2006, 2024). The control-value theory (CVT) provides a framework for understanding how these emotions function within the learning process (Pekrun, 2024). CVT distinguishes between activating and deactivating emotions, which exert complex influences. In EFL listening, positive emotions generally facilitate learning, while negative emotions can trigger concerns about failure that may weaken intrinsic motivation. However, Pekrun (2024) notes that some negative activating emotions, like anxiety, have multifaceted effects. Within CVT, anxiety is increasingly viewed as stemming from uncertainty rather than a simple expectation of failure. Thus, its impact is not straightforward; anxiety can simultaneously foster stronger extrinsic motivation and more rigid, analytical thinking, making its net effect on overall achievement difficult to predict.

Learner autonomy is equally significant in EFL listening, as it centers on the capacity for self-regulated learning, including goal-setting and strategy selection (Yunus and Damayanti, 2024). Unlike more structured writing or reading tasks, listening practice often occurs in informal settings, requiring students to manage authentic language input independently (Yang, 2020). However, this independence seldom develops through learners’ efforts alone without professional guidance. Teacher autonomy support, teaching practices that nurture learners’ initiative rather than controlling the process (Little, 2022), serves as a key external catalyst. It helps students build the internal motivation necessary to maintain consistent listening habits (Alrabai, 2021) and is linked to higher motivation and engagement across language tasks (Reeve, 2009). In listening activities, where negative emotions like anxiety are common, autonomy support can be crucial. By allowing learners to choose materials, set personal goals, and monitor their progress, teachers can help build confidence, mitigate negative emotions, and improve both behavioral and affective engagement (Lai, 2017). Supporting this, Xu and Luo (2024) found that self-regulated listening instruction significantly improved students’ expectations and their valuation of second-language listening comprehension.

Given the unique cognitive demands and attendant emotional challenges of EFL listening, it is essential to understand how academic emotions and autonomy support interact to shape student engagement. Although previous studies have explored these factors in relation to reading and writing, their interplay within listening activities remains far less understood.

## 2 Literature review

This study integrates control-value theory (CVT; Pekrun, 2006) and self-determination theory (SDT; Deci and Ryan, 2000) to examine how teacher-facilitated social contexts interact with students’ affective states to shape learning engagement in EFL listening. Self-determination theory provides the primary framework for understanding how autonomy-supportive environments foster motivation by fulfilling learners’ basic psychological needs for autonomy, competence, and relatedness. Control-value theory then complements this view by specifying how these motivational foundations are subsequently translated into distinct academic emotions, such as enjoyment, anxiety, or boredom, that directly energize or inhibit engaged learning behavior.

### 2.1 Academic emotions and engagement in EFL learning

Academic emotions are crucial in shaping learners’ engagement and achievement (Linnenbrink-Garcia and Pekrun, 2011). Pekrun’s (2006) control-value theory (CVT) posits that emotions such as enjoyment, pride, and anxiety influence students’ engagement and performance by affecting their motivation and cognitive processes. Research confirms that positive academic emotions (e.g., enjoyment, pride) generally foster motivation and engagement, whereas negative emotions (e.g., anxiety, shame, boredom) can impede learning efforts (Pekrun et al., 2007). Extending this framework, Xu et al. (2023) found that academic emotions mediated the relationship between Chinese undergraduates’ control-value appraisals of English and their learning engagement. In EFL listening specifically, the real-time pressure of comprehension and a frequent perceived lack of learner control can intensify these emotional responses, making their influence particularly salient.

Positive emotions, including enjoyment, pride, and hope, are consistently associated with increased engagement in various learning contexts, including EFL (Shao et al., 2019; Li, 2020). For instance, Sadoughi and Hejazi (2021) demonstrated that positive emotions mediate the relationship between teacher support and student engagement. Similarly, Wang et al. (2021) reported that enjoyment and pride are linked to higher EFL self-efficacy. More specifically, enjoyment predicts higher levels of behavioral and affective engagement (Pekrun et al., 2002), while pride in one’s achievements encourages persistence in challenging tasks (Pekrun and Linnenbrink-Garcia, 2012), thereby strengthening engagement.

Conversely, negative emotions such as anxiety, anger, shame, and hopelessness are linked to avoidance behaviors, cognitive overload, and diminished self-efficacy, which hinder effective learning (Wang et al., 2021; Zeidner et al., 2002). Anxiety has been studied extensively due to its prevalence and detrimental impact, especially in listening comprehension (Gregersen and Horwitz, 2002; Horwitz, 2001; Scovel, 1978). In listening, anxiety often originates from the uncertainty of spoken input and real-time processing demands, frequently resulting in disengagement (Elkhafaifi, 2005; Pekrun, 2024). The present study aligns with prior findings, anticipating negative relationships between anxiety, anger, shame, and hopelessness with both behavioral and affective engagement. Boredom, another significant negative emotion, is similarly expected to correlate negatively with engagement, highlighting its potential to divert attention from the listening task.

### 2.2 Autonomy support and its mediating role

While academic emotions significantly influence learning, their effects are moderated by the social-environmental context of the classroom. Autonomy support, defined as instructional practices that foster learners’ sense of volition and willingness to engage (Deci and Ryan, 2000), is widely recognized as a key factor in promoting student engagement. Grounded in self-determination theory (SDT), research indicates that autonomy-supportive teaching enhances student motivation, engagement, and achievement (Reeve, 2012). In EFL contexts, such support is linked to higher motivation, improved communication skills, and stronger engagement, as it helps render learning activities more personally meaningful and enjoyable (Chen and Jang, 2010; Noels et al., 1999; Zarrinabadi et al., 2021). As Zhang et al. (2022) suggest, autonomy support functions as a key social-contextual factor that satisfies students’ basic psychological needs, thereby fostering positive affect and engaged learning behavior.

Critically, it is hypothesized that perceived autonomy support mediates the relationship between academic emotions and learning engagement. Autonomy-supportive teaching can amplify the benefits of positive emotions while buffering the detrimental effects of negative ones. This mediation operates through the interaction between a learner’s affective state and the opportunities for self-direction provided by the teacher. For example, when learners experience pride, an autonomy-supportive environment may allow them to leverage that positive emotion by taking greater ownership of their learning process (Williams and Deci, 1996). Zarrinabadi et al. (2021) similarly found that teacher autonomy support boosted students’ confidence and willingness to use English. Regarding negative emotions, autonomy support can directly counteract disengagement; for instance, by offering more relevant or interesting tasks in response to boredom, a teacher can increase the task’s perceived value and mitigate its negative impact.

### 2.3 Academic emotions, autonomy support, and EFL listening learning

Research increasingly examines the role of academic emotions in engagement, particularly through their interaction with social-environmental factors like autonomy support (Skinner et al., 2009). Within an integrated CVT and SDT framework, instructional factors are not merely external conditions but constitute the social context that shapes the emotional and motivational underpinnings of engagement. As Zhang et al. (2022) argue, the efficacy of autonomy support lies in its capacity to satisfy students’ psychological needs, which subsequently influences their control-value appraisals and emotional experiences. This interplay is especially pertinent in challenging domains like EFL listening, where the transient nature of spoken input can undermine a learner’s sense of control. In such contexts, autonomy-supportive teaching can help maintain engagement over time by fulfilling psychological needs and enhancing perceived control (Reeve and Jang, 2006; Xu and Luo, 2024). When teachers provide opportunities for independent decision-making, they can lower the cognitive and emotional barriers posed by anxiety or boredom, thereby sustaining student engagement.

## 3 The present study

Although academic emotions and autonomy support have garnered increasing attention in EFL research, their interrelationships and combined influence on engagement in EFL listening remain underexplored. To address this gap, the present study adopts an integrated CVT and SDT framework to examine how teacher-facilitated social contexts interact with learners’ affective states. Specifically, it investigates how academic emotions (both positive and negative) impact behavioral and affective engagement in listening tasks. Furthermore, it examines the psychological mechanism through which perceived teacher autonomy support mediates these relationships.

Compared to other language skills, listening often demands real-time comprehension without visual cues or opportunities for review, rendering it particularly cognitively and emotionally demanding. In this high-pressure context, teacher autonomy support serves as a critical external resource that can aid students in regulating their emotional responses. Consequently, this study seeks to provide practical insights into how instruction can support learners’ internal efforts to maintain engagement in EFL listening.

RQ1: To what extent are positive and negative academic emotions associated with engagement in EFL listening?

RQ2: How does perceived teacher autonomy support mediate the relationship between academic emotions and listening engagement?

Based on the research questions and elucidation above, we had following hypotheses.

*H1*: Positive academic emotions (enjoyment, hope, pride) are positively associated with both behavioral and affective engagement in EFL listening.

*H2*: Negative academic emotions (anger, anxiety, shame, hopelessness, boredom) are negatively associated with engagement in EFL listening.

*H3*: Perceived autonomy support mediates the relationship between academic emotions and engagement, providing students with the agency to leverage positive affect and mitigate the impact of negative affect.

## 4 Method

### 4.1 Participant

219 English university learners (mean age: 19.1 ± 0.8 years; 41 males) participated in the present study. They were all required to attend English listening courses every semester during the first two years of a four-year undergraduate program. Most of the learners were freshmen (85.4%). The rest were sophomores (12.3%) and juniors (2.3%).

### 4.2 Measurements

The questionnaire was divided into four sections. The first section collected demographic information from the learners. The second section gauged academic emotions pertaining to English listening learning. The academic emotions questionnaire used a five-point scale and was adapted from the Academic Emotions Questionnaire-Short version (AEQ-S), an abbreviated version of the AEQ (Bieleke et al., 2021). To ensure linguistic and conceptual equivalence, a rigorous translation and back-translation procedure was followed. The original English items were first translated into Chinese by two bilingual researchers and then back-translated into English by another independent translator to check for accuracy. During this process, the items were contextualized for the English listening context. The final scale encompassed 31 items on a five-point scale designed to measure a range of academic emotions: enjoyment (4 items), hope (4 items), pride (3 items), anger (4 items), anxiety (4 items), shame (4 items), hopelessness (4 items), and boredom (4 items). Confirmatory factor analyses confirmed that all the academic emotions scales demonstrated adequate structural validity and reliability (refer to Table 1). The third section measured perceived autonomy support from teachers. Items from the Perceived Learning Climate Scale (Williams and Deci, 1996) were adapted to fit the English listening context using a five-point scale, and 5 items were retained (see Table 1 for evidence of validity and reliability). The fourth section measured two facets of learning engagement in listening (behavioral engagement with five items and affective engagement with five items, both on a five-point scale). The items were adapted from Skinner et al. (2009) for the English listening context.

**Table 1:** Reliability and validity for measurements.

Construct	CFI	NFI	IFI	SRMR	AVE	α
Enjoyment	0.92	0.92	0.92	0.05	0.57	0.81
Hope	0.98	0.98	0.98	0.09	0.72	0.91
Pride	1	1	1	0	0.53	0.74
Anger	1	0.99	1	0.01	0.51	0.80
Anxiety	1	0.99	1	0.01	0.52	0.81
Shame	0.99	0.98	0.99	0.03	0.49	0.78
Hopeless	0.99	0.99	0.99	0.02	0.56	0.82
Boredom	0.94	0.94	0.95	0.03	0.55	0.83
BE	0.93	0.92	0.93	0.04	0.54	0.85
AE	0.97	0.96	0.97	0.03	0.62	0.89
Autonomy	0.98	0.98	0.99	0.02	0.58	0.87

BE: behavioral engagement, AE: affective engagement, CFI: comparative fit index, NFI: Bentler-Bonett Normed Fit Index, IFI: Bollen’s Incremental Fit Index, AVE: average variance extracted.

### 4.3 Procedure

In this study, data were collected through a questionnaire administered to students during their regular English listening classes. Participants received an introduction to the study, its purpose, and ethical considerations, including the voluntary nature of participation and the confidentiality of their responses. They were given 20–30 min to complete the survey, ensuring they had sufficient time to respond accurately. To mitigate potential biases, participants were assured that their responses would not affect their academic evaluations or relationships with instructors. The data were analyzed using JASP (Version 19.3.0). We first examined the descriptive statistics (means, standard deviations, skewness, and kurtosis) for all study variables to assess distributional properties. Bivariate Pearson correlation coefficients were then computed to explore preliminary associations among academic emotions, autonomy support, and EFL listening engagement. To test the hypothesized mediation model, specifically, whether autonomy support mediates the relationship between academic emotions (predictors) and learning engagement in EFL listening (outcome), we employed the mediation analysis module in JASP, which is based on the lavaan package and uses maximum likelihood estimation. The model included eight academic emotions (enjoyment, hope, pride, anger, anxiety, shame, hopelessness, boredom) as predictors, autonomy support as the mediator, and both behavioral engagement and affective engagement in EFL listening as outcome variables. We specified a standard mediation model with all direct and indirect paths estimated simultaneously. Three key statistical assumptions were verified prior to analysis: (1) Linear relationships: Pearson correlations confirmed linear associations between predictors, the mediator, and outcomes; (2) Multivariate normality: Skewness (−0.57 to 0.80) and kurtosis (−0.86 to 0.34) values fell within acceptable ranges (Kline, 2015); (3) No multicollinearity: Variance Inflation Factor (VIF) values for all < 10, indicating no significant multicollinearity (Hair et al., 2019). Statistical significance was determined at *p* < 0.05, with *p* < 0.01 and *p* < 0.001 also reported where applicable. To address Type I error inflation due to multiple comparisons, two complementary strategies were adopted: (1) 95% bias-corrected bootstrap confidence intervals (1,000 resamples) were reported for all direct and indirect effects, with non-zero intervals indicating significance; (2) Effect sizes (f^2^) and confidence intervals were emphasized alongside *p*-values, avoiding overreliance on statistical significance alone (Benjamin et al., 2018). In addition to standardized coefficients, the proportion of variance explained (R^2^) was reported for the outcome variables.

## 5 Results

### 5.1 Descriptive statistics

Descriptive statistics are presented in Table 2. Mean scores for all variables fell within a moderate range (2.32–3.89 on a 5-point scale). Participants reported moderate levels of positive emotions (enjoyment, hope, pride), negative emotions (anger, anxiety, shame, hopelessness, boredom), learning engagement, and perceived teacher autonomy support. Normality tests indicated that skewness and kurtosis values for all major variables fell within acceptable limits, confirming the data were suitable for parametric analyses.

**Table 2:** Descriptive Statistics on all variables.

Variable	Mean	SD	Skewness	Kurtosis
Enjoyment	3.74	0.82	−0.03	−0.86
Hope	3.39	1.00	0.11	−0.77
Pride	3.80	0.85	−0.27	−0.71
Anger	2.07	0.89	0.80	0.34
Anxiety	2.91	1.04	−0.06	−0.59
Shame	2.69	0.97	0.17	−0.49
Hopeless	2.42	1.00	0.29	−0.63
Boredom	2.32	0.94	0.38	−0.63
Autonomy support	3.83	0.85	−0.57	0.26
Behavioral engagement	3.89	0.74	−0.14	−0.74
Affective engagement	3.71	0.87	−0.40	−0.04

### 5.2 Correlation among the factors

As shown in Table 3, all positive emotions correlated positively with autonomy support and learning engagement. The negative emotions of anger, shame, hopelessness, and boredom correlated negatively with autonomy support and engagement. Anxiety showed a negative but non-significant correlation with autonomy support (*r* = −0.13).

**Table 3:** Correlation matrix on all variables.

Variable	1	2	3	4	5	6	7	8	9	10
1. Enjoyment	——									
2. Hope	0.76^***^	——								
3. Pride	0.79^***^	0.75^***^	——							
4. Anger	−0.62^***^	−0.59^***^	−0.49^***^	——						
5. Anxiety	−0.40^***^	−0.69^***^	−0.37^***^	0.61^***^	——					
6. Shame	−0.42^***^	−0.67^***^	−0.36^***^	0.66^***^	0.82^***^	——				
7. Hopeless	−0.53^***^	−0.73^***^	−0.48^***^	0.71^***^	0.78^***^	0.83^***^	——			
8. Boredom	−0.66^***^	−0.58^***^	−0.49^***^	0.80^***^	0.53^***^	0.61^***^	0.67^***^	——		
9. Autonomy support	0.59^***^	0.48^***^	0.60^***^	−0.33^***^	−0.13	−0.21^***^	−0.26^***^	−0.43^***^	——	
10. Behavioral engagement	0.72^***^	0.61^***^	0.66^***^	−0.53^***^	−0.32^***^	−0.32^***^	−0.43^**^	−0.55^***^	0.62^***^	——
11. Affective engagement	0.78^***^	0.62^***^	0.69^***^	−0.56^***^	−0.29^***^	−0.35^***^	−0.47^***^	−0.62^***^	0.75^***^	0.74^***^

^*^p < 0.05; ^**^p < 0.01; ^***^p < 0.001.

### 5.3 How learning autonomy support mediated the academic emotions and listening learning engagement

The mediation analyses revealed that academic emotions differentially predicted behavioral and affective listening engagement. The model explained 61.0% of the variance in behavioral engagement and 75.7% of the variance in affective engagement, indicating strong explanatory power.

The results showed that enjoyment had a direct positive effect on both behavioral and affective engagement. Moreover, Autonomy support fully mediated the relationship between pride and both engagement facets, as well as between boredom and both engagement facets. Autonomy support mediated the relationship between anxiety and affective engagement. Direct effects were confirmed for anger and shame (on behavioral engagement) and hopelessness (on affective engagement) (see Table 4 for details).

**Table 4:** Mediating effect testing of learning autonomy support for the relationship between academic emotions and listening learning engagement.

Predictor → Outcome	Total effects	Direct effects	Indirect effects via autonomy support
	[95%CI]	[95%CI]	[95%CI]
Enjoyment → BE	0.34^**^ **[0.16, 0.53]**	0.30^***^ **[0.12, 0.48]**	0.05 [−0.01, 0.11]
Hope → BE	0.15 [−0.06, 0.36]	0.10 [−0.10, 0.30]	0.05 [−0.02, 0.12]
Pride → BE	0.21^*^ **[0.05, 0.37]**	0.10 [−0.06, 0.26]	0.10^**^ **[0.04, 0.17]**
Anger → BE	−0.13 [−0.30, 0.04]	−0.16^*^ **[−0.32, −0.00]**	0.03 [−0.02, 0.08]
Anxiety → BE	0.04 [−0.13, 0.21]	−0.03 [−0.20, 0.13]	0.07^*^ **[0.01, 0.14]**
Shame → BE	0.15 [−0.04, 0.33]	0.18^*^ **[0.01, 0.36]**	−0.04 [−0.10, 0.03]
Hopeless → BE	−0.02 [−0.20, 0.17]	−0.06 [−0.21, 0.10]	0.04 [−0.02, 0.10]
Boredom → BE	−0.13 [−0.29, 0.03]	−0.06 [−0.21, 0.10]	−0.08^*^ **[−0.14, −0.02]**
Enjoyment → AE	0.43^***^ **[0.27, 0.59]**	0.36^***^ **[0.22, 0.49]**	0.08 [−0.02, 0.17]
Hope → AE	0.08 [−0.11, 0.27]	0.00 [−0.15, 0.16]	0.08 [−0.03, 0.19]
Pride → AE	0.19^**^ **[0.05, 0.34]**	0.03 [−0.10, 0.16]	0.16^***^ **[0.07, 0.25]**
Anger → AE	−0.06 [−0.21, 0.09]	−0.11 [−0.23, 0.02]	0.05 [−0.04, 0.13]
Anxiety → AE	0.19^*^ **[0.05, 0.35]**	0.08 [−0.06, 0.21]	0.12^*^ **[0.03, 0.20]**
Shame → AE	0.05 [−0.12, 0.21]	0.10 [−0.04, 0.24]	−0.05 [−0.15, 0.04]
Hopeless → AE	−0.10 [−0.27, 0.07]	−0.16^*^ **[−0.30, −0.02]**	0.06 [−0.03, 0.15]
Boredom → AE	−0.20^*^ **[−0.35, −0.06]**	−0.09 [−0.21, 0.04]	−0.12^**^ **[−0.20, −0.04]**

^*^p < 0.05; ^**^p < 0.01; ^***^p < 0.001. Bold values denote statistically significant effects.

Effect sizes (*f^2^*) were calculated to quantify practical significance (Cohen, 1988): small effects (*f^2^* = 0.02–0.14), medium effects (*f^2^* = 0.15–0.34), and large effects (*f^2^* ≥ 0.35). As shown in Table 4, the mediating effect of autonomy support on pride → behavioral engagement had a small-to-medium effect size (*f^2^* = 0.12), while the mediating effect on boredom → affective engagement was also small-to-medium (*f^2^* = 0.11). The direct effect of enjoyment on affective engagement was medium (*f^2^* = 0.23), indicating meaningful practical impacts. The results partially supported H1 and H2. For H1, enjoyment and pride were significant predictors of engagement, but hope was not. For H2, boredom and hopelessness showed the expected negative associations with engagement, while anxiety and shame exhibited complex (occasionally positive) relationships, suggesting the impact of negative affect varies by emotion type. H3 was supported: autonomy support mediated the relationship between specific academic emotions (pride, anxiety, boredom) and engagement, facilitating positive affect and buffering negative affect.

## 6 Discussion

This study aimed to explore the roles of academic emotions and autonomy support in EFL listening learning engagement. The findings directly address our two research questions. First, academic emotions predict listening engagement in different ways. Second, teacher autonomy support serves as a critical mediator that either facilitates the positive impact of emotions like pride or buffers the negative effects of boredom and anxiety. Based on the integrated framework of CVT and SDT, this study elucidates the social-psychological mechanisms that drive task engagement in the challenging context of EFL listening.

### 6.1 The role of academic emotions in EFL listening engagement

Our results highlight the close relationship between both positive and negative emotions and students’ engagement in EFL listening. Positive emotions like enjoyment, hope, and pride were positively correlated with both behavioral and affective engagement, aligning with previous studies that point to the benefits of positive feelings in learning (Brackett et al., 2012; Pekrun, 2006; Pekrun and Linnenbrink-Garcia, 2012; Sadoughi and Hejazi, 2021; Wang et al., 2021). Among these emotions, enjoyment was found to have a direct influence on both behavioral and affective engagement in listening. In other words, students are more likely to participate actively and stay engaged in listening tasks when they experience pleasure during the process.

Interestingly, pride, although traditionally associated with success in academic tasks (Pekrun et al., 2009; Wang et al., 2021), was found to have its impact on engagement mediated by the perceived autonomy support from teachers. From the perspective of SDT, pride can be viewed as a psychological signal that a student’s need for competence has been satisfied through successful listening performance. However, our findings suggest that this sense of competence (pride) requires the fulfillment of the need for autonomy to effectively drive engagement. This implies that for learners to translate a sense of pride in their EFL listening into engagement, they need a supportive learning environment that cultivates a sense of control and ownership over their learning process (Little, 1995). According to previous studies, an autonomy-supportive EFL learning environment can empower students by decreasing threats and increasing intrinsic incentives (Alrabai, 2020; Black and Deci, 2000). It highlights the pivotal role of autonomy support in translating students’ intrinsic motivation and pride into actual engagement, a point which may be particularly significant in EFL listening, where learners often face comprehension difficulties (Goh, 2014; Vandergrift and Goh, 2012).

In contrast to the effects of positive emotions, negative emotions such as anger, boredom, and hopelessness were found to be correlated with lower levels of student engagement. This result is consistent with previous research, which has shown how negative emotions can reduce motivation and interfere with learning outcomes (Pekrun et al., 2007; Wang et al., 2021; Zeidner et al., 2002). These emotions can create a mental barrier that prevents students from focusing on learning tasks. For example, boredom showed a strong negative correlation with both behavioral and affective engagement. That is, when students perceive listening tasks as repetitive, they are more likely to become distracted from the learning process. This highlights the unique challenge of EFL listening, where learners must concentrate on fast-moving spoken input without the opportunity to pause or review. Therefore, staying focused is especially challenging due to the nature of spoken language, the complexity of the language, and background noise in the classroom environment (Hardiyanto et al., 2021; Vandergrift, 2007).

Additionally, anger and shame were found to directly affect behavioral engagement, while hopelessness was more strongly related to affective engagement. The relationship between anxiety and engagement was complex in our study. While students with lower levels of anxiety generally perform better in EFL listening (Gilakjani and Sabouri, 2016), our results showed that anxiety did not exert a significant direct negative impact on engagement. Instead, its influence was primarily manifested through an indirect path. This indicates that the effect of anxiety on listening engagement is not inherently debilitating but is highly dependent on environmental factors, a mechanism that will be further explored in the next section regarding the role of teacher support. These findings suggest that different negative emotions may uniquely influence how learners participate in listening tasks. For example, learners who feel ashamed or overwhelmed may lose interest or motivation easily, while anger could lead to resistance or disengagement from the task itself. The varied effects of negative emotions highlight the need for practical support strategies in EFL listening classrooms. When students struggle to understand spoken input, frustration can build quickly and lead to disengagement (Elkhafaifi, 2005). Moreover, students with lower levels of anxiety tend to perform better in EFL listening, suggesting that emotional support and guidance from teachers play an important role in helping learners improve their listening skills (Gilakjani and Sabouri, 2016).

### 6.2 The mediating role of autonomy support

The results also show that autonomy support from teachers acts as a mediator in several of the relationships between academic emotions and engagement. This finding is in line with self-determination theory (Deci and Ryan, 2000), which posits that autonomy support is crucial for cultivating intrinsic motivation and engagement. Furthermore, our findings also extend those of Xu et al. (2023), who found that control-value appraisals serve as a mediator between students’ academic emotions and engagement in EFL classrooms. In the framework of SDT, pride can be interpreted as a psychological signal that a student’s need for competence has been satisfied. Our results show that when students perceive their teachers’ support for their autonomy, such as through the provision of choices and opportunities for initiative, this sense of competence is effectively validated and fosters active engagement. Conversely, autonomy support reduces feelings of boredom by allowing students to find personal relevance and value in the listening materials, thereby addressing the cause of disengagement.

Moreover, autonomy support also played a mediating role between anxiety and affective engagement. While anxiety is often regarded as a negative force in EFL listening (Vandergrift and Goh, 2012), our findings revealed a significant positive indirect effect alongside a non-significant direct effect. This suggests that teacher autonomy support may function as a psychological buffer against the negative effects of anxiety. When learners perceive that their teacher supports their autonomy, they may view their anxiety less as a barrier. Instead, they are better able to manage anxiety and become emotionally engaged in the learning process. This finding aligns with Zarrinabadi et al.’s study (2021), which demonstrated that teachers’ autonomy support can enhance students’ perceived competence in using English and effectively transform a potentially negative emotional state into a driver for consistent task engagement.

### 6.3 Limitations and future research

While this study offers valuable insights into the relationships between academic emotions, autonomy support, and EFL listening engagement, it also has several limitations that should be noted. First, the cross-sectional design limits causal claims. Longitudinal research is needed to examine how students’ emotions and engagement evolve over time. Second, this study focuses on English listening learning. Future research might explore how emotional and motivational factors interact in speaking, reading, or writing learning, where the cognitive demands and classroom dynamics may differ. Third, our study did not account for the influence of teacher behaviors. Teachers’ instructional styles, emotional expressions, and classroom management practices may shape students’ emotional experiences and perceived autonomy support.

## 7 Conclusion

This study demonstrates that academic emotions and teacher autonomy support are integral to student engagement in EFL listening. The findings reveal that while enjoyment directly promotes engagement, the effects of pride, boredom, and anxiety are largely channeled through teacher autonomy support. Specifically, a supportive classroom climate provides the psychological security required for students to maintain focus despite the cognitive pressures of real-time comprehension. Practically, these results suggest that listening instruction should prioritize student self-governance. By shifting from a directive approach to one that incorporates student choice and initiative, instructors can better assist learners in navigating emotional challenges and sustaining their commitment to the listening process.

## Data Availability

The raw data supporting the conclusions of this article will be made available by the authors, without undue reservation.
